# Lunate Dislocation and Basic Wrist Kinematics

**Published:** 2016-08-24

**Authors:** Maxwell Wingelaar, Patrick Newbury, Nicholas S. Adams, Andrew J. Livingston

**Affiliations:** ^a^Michigan State University College of Human Medicine, Grand Rapids, Mich; ^b^Grand Rapids Medical Education Partners Plastic and Reconstructive Surgery Residency, Grand Rapids, Mich; ^c^Plastic Surgery Specialists, Grand Rapids, Mich

**Keywords:** lunate dislocation, wrist kinematics, Mayfield classifications, carpal instability, progressive perilunate instability

## DESCRIPTION

**Figure 1:**
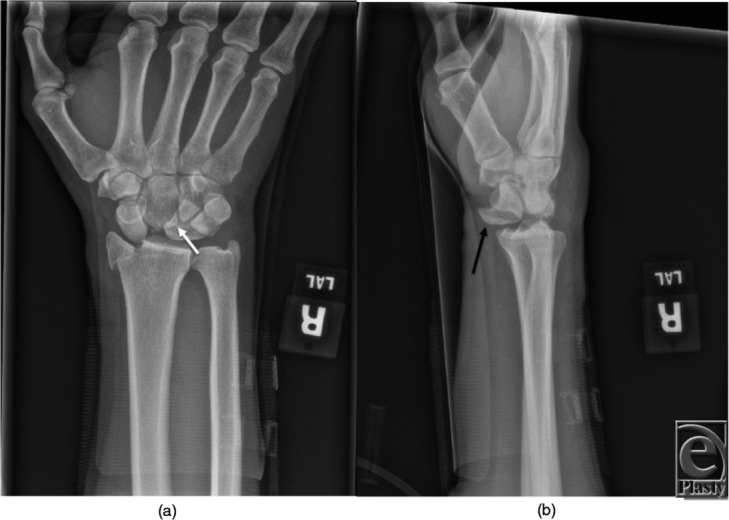
(a) Anteroposterior view of the right wrist demonstrating transradial styloid lunate dislocation. The lunate is seen overlapping the capitate, creating the classic “piece-of-pie” sign (*white arrow*). (b) Lateral view of the right wrist demonstrating lunate dislocation. The lunate is displaced and angulated out of the lunate fossa, producing the “spilt teacup” appearance (*black arrow*).

A 57-year old man presented to the emergency department for right wrist pain following a motorcycle crash. Physical examination revealed right wrist edema, pain with passive and active range of motion, and tenderness to palpation over the dorsal and volar wrist. Sensation was mildly diminished in the right thumb and the index and long fingers. X-rays of the right wrist were done (Figs 1*a* and 1*b*).

## QUESTIONS

1. **What are the basic kinematic forces that act on the lunate?**
2. **What is the Mayfield classification, and what stage is this patient's wrist injury?**
3. **What are the immediate- and long-term concerns following lunate dislocation?**
4. **What treatment options exist for the long-term sequelae of lunate dislocations?**

## DISCUSSION

The wrist is a complex joint comprising the radius, ulna, and 8 carpal bones organized into 2 rows, the proximal row (PR) containing the scaphoid, lunate, pisiform, and triquetrum and the distal row (DR), comprising the trapezium, trapezoid, capitate, and hamate. It undergoes a complex degree of movement during day-to-day tasks, with, in general, greater motion occurring in the PR than in the DR. During ulnar deviation and extension, the PR extends while the DR rotates ulnarly. The opposite is true with radial deviation and flexion. When a compressive load is applied to the wrist, scaphoid flexion occurs. This is due to its volar orientation in the wrist. In contrast, the triquetrum extends during compression. The lunate lies between the scaphoid and triquetrum and is intimately attached to both through the scapholunate (SL) and lunotriquetral (LT) intercarpal ligaments. These 2 major intercarpal attachments maintain the lunate in a state of balance between the opposing forces. When there is disruption of the SL or LT ligaments, the balance is lost and the lunate is dominated by the remaining intercarpal relationship. Normally, the angle between the scaphoid and lunate is less than 60°.^1^ Disruption of the SL ligament releases the scaphoid from the balanced PR, moving it into flexion and increasing the SL angle. The lunate then follows the triquetrum into unopposed extension. Disruption of the LT ligament decreases the SL angle as the lunate loses its extension influence from the triquetrum.

**Figure 2:**
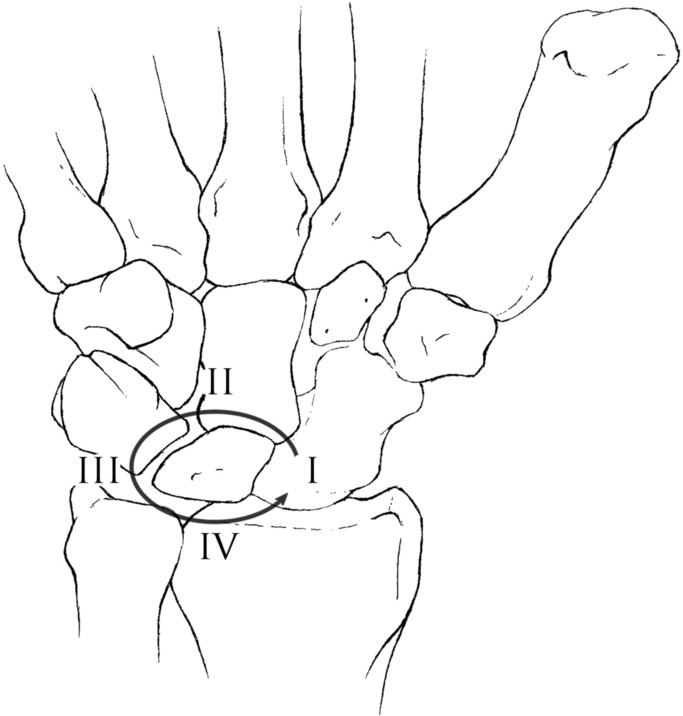
Right volar wrist demonstrating the pattern of progressive perilunate instability described by Mayfield *et al*.^2^ Illustration provided by Lindsey A. Behrend, BS.

**Figure 3:**
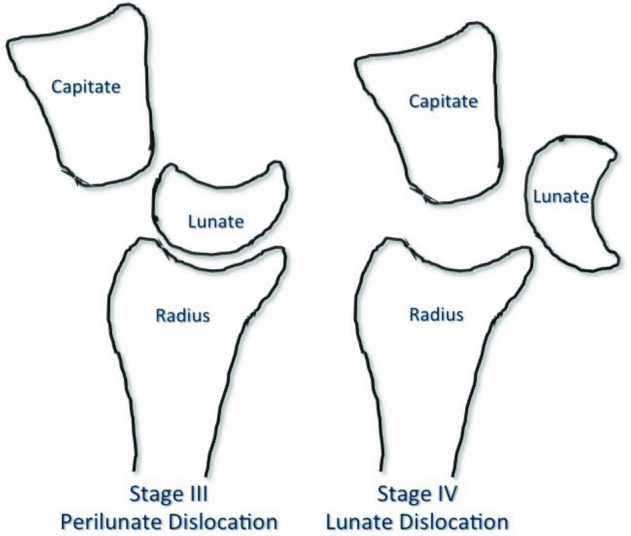
Diagram illustrating perilunate and lunate dislocations. Note the position of the lunate relative to the lunate fossa of the distal radius. The lunate remains in the fossa in perilunate dislocations.

**Figure 4:**
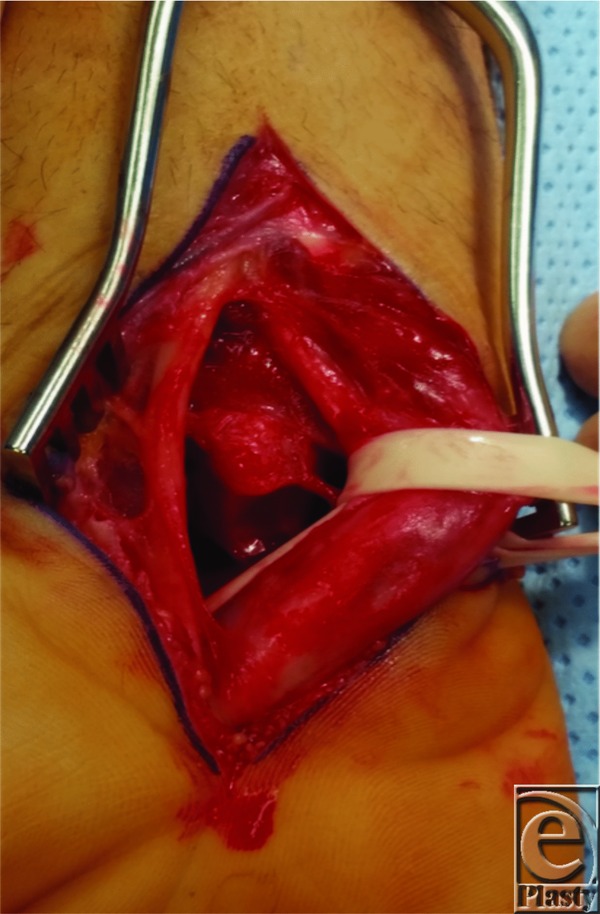
Volar exposure of the wrist. Note the lunate dislocated into the carpal tunnel.

In 1980, Mayfield *et al*^2^ published their classification system of progressive perilunate instability. This system described the propagation of forces leading to the progression of carpal dislocation. It consists of 4 progressive stages ranging from I to IV (Fig 2). Stage I is characterized by disruption of the SL joint, most notably the SL interosseous ligament. This may be visible as an intercarpal space of greater than 2 to 3 mm between the lunate and scaphoid on the anteroposterior radiograph (Terry Thomas sign). Stage II progresses past stage I to include capitolunate joint subluxation. Stage III continues on disrupting the LT joint and ligament. This most often results in dorsal dislocation of the carpus, known as perilunate dislocation. The fourth stage progresses to disruption of the radiolunate joint and represents lunate dislocation (Figs 1*a*, 1*b*, and 3). The lunate leaves its normal articulation with the radius and may dislocate into the carpal tunnel through the space of Poirier, a weakness in the floor of the carpal tunnel (Fig 4). The short radiolunate ligament frequently retains the volar lunate from complete translocation into the carpal tunnel.^2^^,^^3^ The patient described here represents stage IV lunate dislocation following a motorcycle crash.

Urgent closed reduction should be performed if immediate surgical repair is not planned. There are a multitude of surgical techniques used for correction of lunate dislocations. Open reduction with internal fixation (ORIF) with ligament repair utilizing dorsal, volar, or combined approaches is one of the most commonly employed techniques. ORIF involves reducing and fixating the dislocated lunate using Kirschner wire (K-wire) fixation. Reduction can be assisted by the use of 0.062-in K-wires as joysticks to align the carpus. K-wires are then inserted through the scaphoid and lunate to fixate the SL diastasis. Next, the SL ligament can be repaired acutely using suture anchors. The LT interosseous ligament may also be repaired using similar techniques; however, limited data exist validating this additional step. When comparing dorsal or volar approaches, no differences in outcomes have been revealed.^4^ However, the volar approach may be preferred when carpal tunnel release is indicated.^5^ Carpal tunnel release should be done in the presence of median nerve symptoms but is not needed in their absence. Even with early repair, many patients often experience poor long-term results following lunate dislocations. Degenerative arthritis, loss of grip strength, SL advanced collapse, median nerve irritation, and chronic wrist instability are common postinjury sequelae. The prognoses of lunate and perilunate dislocations are similar, and both types of injuries place patients at an increased risk of developing these long-term problems.^6^

By utilizing ORIF and ligamentous repair in an expeditious manner, the patient's chances of developing long-term sequelae can be reduced. However, most patients may still experience some degree of decreased grip strength and remain at an increased risk of developing degenerative arthritis. Further surgical correction is often required to treat these sequelae, including SL ligament reconstruction, PR carpectomy, total wrist arthrodesis, or total wrist arthroplasty. Each option has advantages and disadvantages, but when there is loss of the articular surface in the lunate fossa of the radius or the proximal capitate, PR carpectomy should be avoided.^7^^,^^8^ Nonsurgical management of chronic symptoms such as nonsteroidal anti-inflammatory drugs, hot and cold therapy, corticosteroid injections, and hand therapy have proven helpful in some cases.^6^

Lunate dislocation progresses in a reproducible pattern. Early reduction and repair are paramount to optimize outcomes. However, the risk of developing degenerative arthritis remains high.
